# Editor's Note on ‘Inhibition of cyclin D1 expression by androgen receptor in breast cancer cells—identification of a novel androgen response element’

**DOI:** 10.1093/nar/gkaf915

**Published:** 2025-09-12

This an editor's note regarding: Marilena Lanzino, Diego Sisci, Catia Morelli, Cecilia Garofalo, Stefania Catalano, Ivan Casaburi, Claudia Capparelli, Cinzia Giordano, Francesca Giordano, Marcello Maggiolini, Sebastiano Andò, Inhibition of cyclin D1 expression by androgen receptor in breast cancer cells—identification of a novel androgen response element, *Nucleic Acids Research*, Volume 38, Issue 16, 1 September 2010, Pages 5351–5365, https://doi.org/10.1093/nar/gkq278

In April 2025, the Editors were alerted to potential duplication of image panels within the NAR article, specifically between Figs. 8C and 8E, and between Figs. 6B and 8E. Additionally, another potential duplication was identified between Fig. 5C of the NAR article and Fig. 5C of a separate publication by the same authors [1]. These concerns have also been raised on PubPeer (https://pubpeer.com/publications/04ED7DC17454ABBA537AF087C41379#3).

The Editor contacted the authors, who no longer have access to the original data because the experiments were conducted over 15 years ago. However, the authors provided images from preliminary experiments conducted at the time (Supplementary File 1). The authors acknowledge the possibility of an error during figure assembly but stand by their results which have since been independently confirmed by other research groups [2–5].

The Editors carefully reviewed the images and found no clear evidence of panel duplication.

However, while the issues described above may not affect the results or conclusion of the study, in the absence of original data, the Editors advise readers to examine Figs. 5C, 6B, 8C and 8D with care.

## Supplementary Material

gkaf915_Supplemental_File

## Supplementary data

Supplementary data is available at NAR online.
