# Supplementary material for: Editor's Note on ‘Inhibition of cyclin D1 expression by androgen receptor in breast cancer cells—identification of a novel androgen response element’
Source: Nucleic Acids Res. 2025 Sep 12;53(17):gkaf915. doi: 10.1093/nar/gkaf915 (PMC12445689; doi:10.1093/nar/gkaf915)

9/7/07 | ChiP 0-1-2-4h DHT | MCF7/B IP:AR

- Starvation 1 Day.
- Stimolazione 1-2-4h in 2% DCC

IP: AR

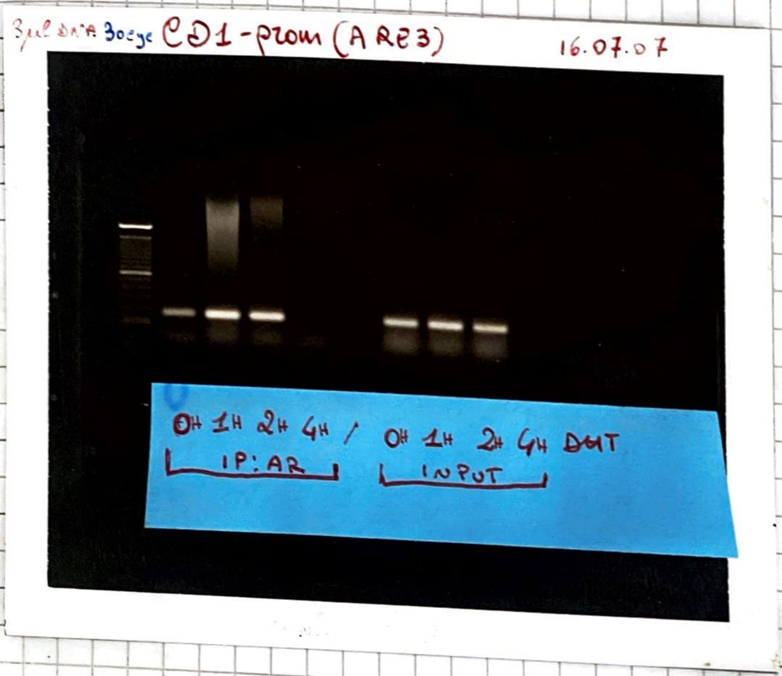

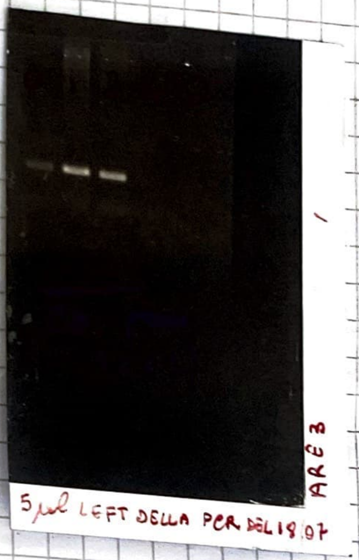

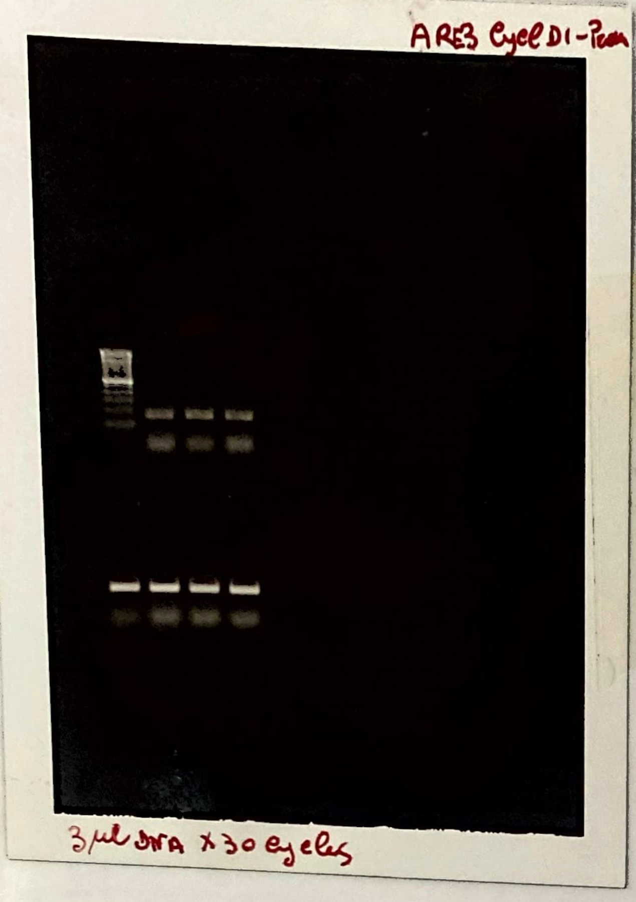
ARE3 Cycl D1-Pam
3µl DNA x 30 cycles

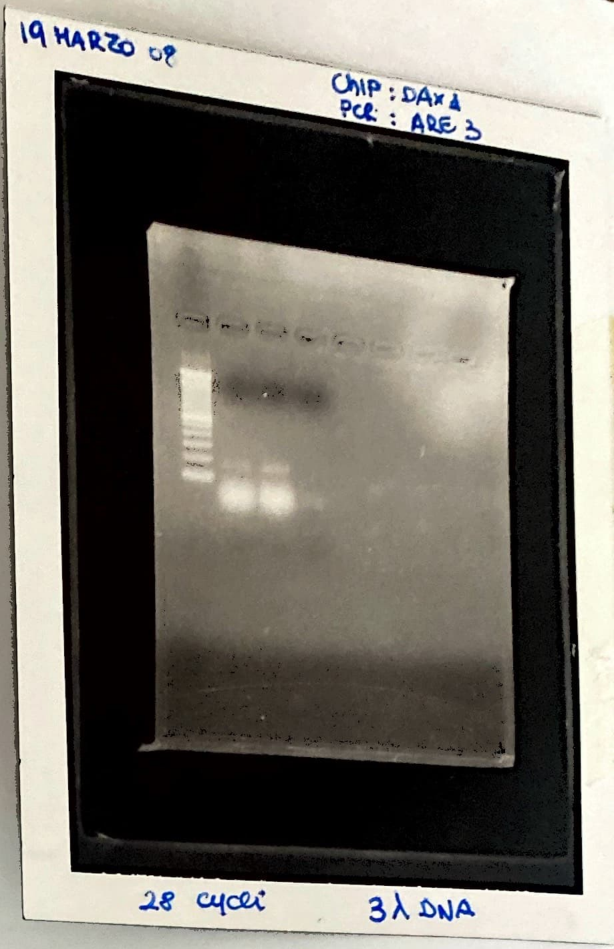
19 MARZO 08
ChIP : DAX1
PCR : ARE 3
28 cycli
3λ DNA

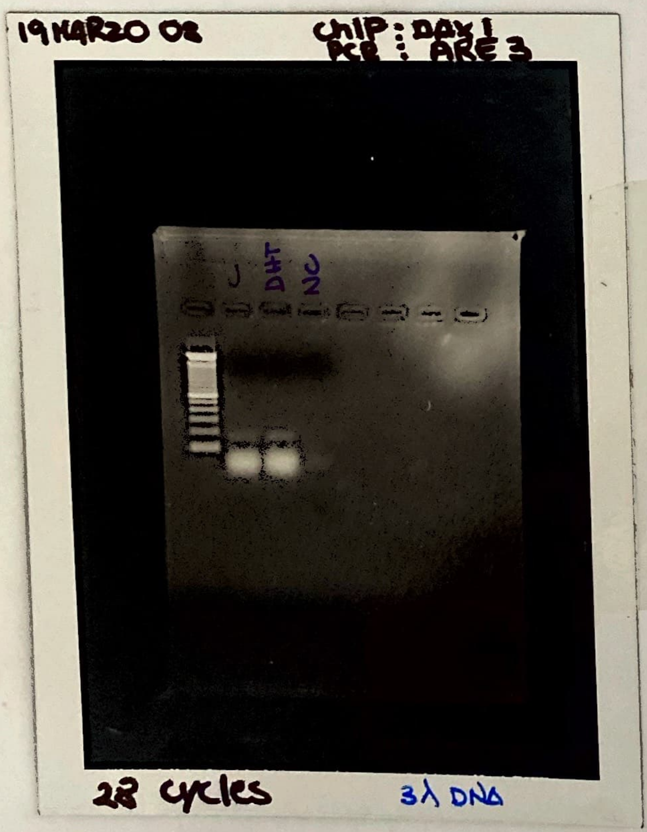
19 MARZO 08
ChIP : DAX1
PCR : ARE 3
DHT
NC
28 cycles
3λ DNA

15/01/09 DAPA HDAC 1/3 on CD1 prom ARE3 Ok

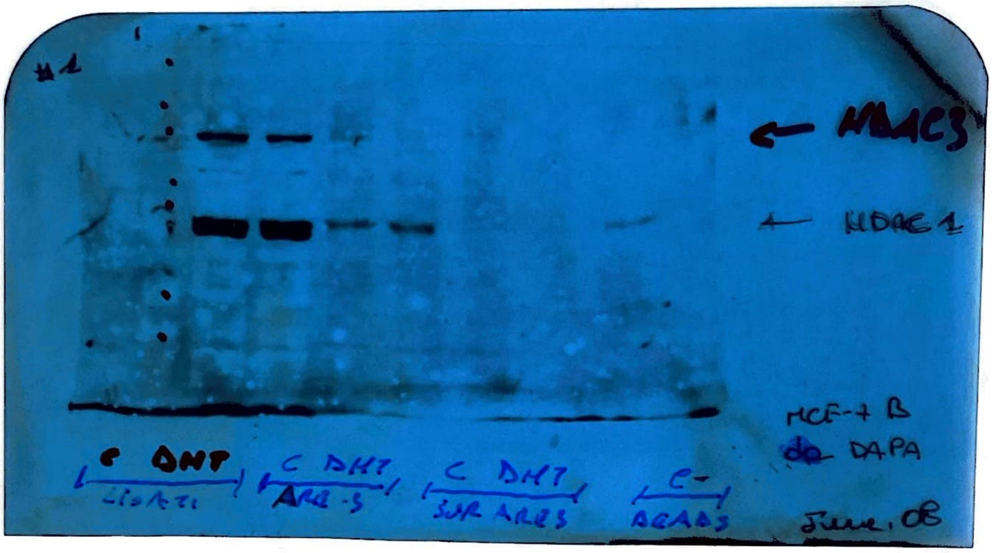

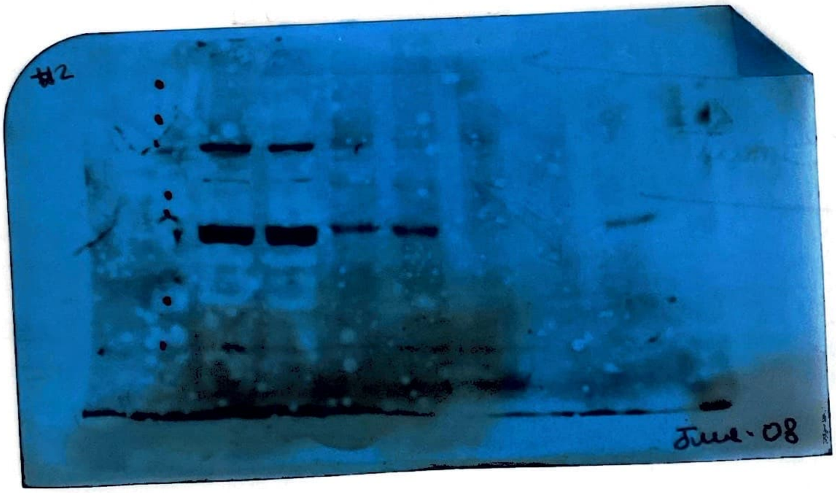

Supplement: gkaf915_Supplemental_File [file gkaf915_supplemental_file.pdf]
